# Food Intolerances, Food Allergies and IBS: Lights and Shadows

**DOI:** 10.3390/nu16020265

**Published:** 2024-01-16

**Authors:** Andrea Pasta, Elena Formisano, Francesco Calabrese, Maria Corina Plaz Torres, Giorgia Bodini, Elisa Marabotto, Livia Pisciotta, Edoardo Giovanni Giannini, Manuele Furnari

**Affiliations:** 1 Gastroenterology Unit, Department of Internal Medicine, University of Genoa, 16132 Genoa, Italy; andreapasta93@gmail.com (A.P.); calabrese.francesco.93@gmail.com (F.C.); maco.plaz87@gmail.com (M.C.P.T.); giorgia.bodini@unige.it (G.B.); elisa.marabotto@unige.it (E.M.); egiannini@unige.it (E.G.G.); 2 Dietetics and Clinical Nutrition Unit, Department of Internal Medicine, University of Genoa, 16132 Genoa, Italy; elena.formisano@hsanmartino.it (E.F.); livia.pisciotta@unige.it (L.P.); 3 IRCCS Ospedale Policlinico San Martino, 16132 Genoa, Italy

**Keywords:** food intolerances, irritable bowel syndrome, gastrointestinal physiology, dietary management, low-FODMAP diet

## Abstract

This narrative review delves into the intricate relationship between irritable bowel syndrome (IBS) and food intolerances. IBS, a chronic functional gastrointestinal disorder, is characterized by symptoms like abdominal pain and altered bowel habits. The prevalence of IBS has increased globally, especially among young adults. Food and dietary habits play a crucial role in IBS management. About 85–90% of IBS patients report symptom exacerbation linked to specific food consumption, highlighting the strong connection between food intolerances and IBS. Food intolerances often exhibit a dose-dependent pattern, posing a challenge in identifying trigger foods. This issue is further complicated by the complex nature of gastrointestinal physiology and varying food compositions. This review discusses various dietary patterns and their impact on IBS, including the low-FODMAP diet, gluten-free diet, and Mediterranean diet. It highlights the importance of a personalized approach in dietary management, considering individual symptom variability and dietary history. In conclusion, this review emphasizes the need for accurate diagnosis and holistic management of IBS, considering the complex interplay between dietary factors and gastrointestinal pathophysiology. It underlines the importance of patient education and adherence to treatment plans, acknowledging the challenges posed by the variability in dietary triggers and the psychological impact of dietary restrictions.

## 1. Introduction

Irritable bowel syndrome (IBS) is a chronic functional gastrointestinal disorder characterized by symptoms like abdominal pain and altered bowel habits [1]. This condition involves a complex interplay of gut–brain interactions, leading to dysmotility and visceral hypersensitivity, and environmental factors, including diet and psychological distress (i.e., anxiety, depression, or somatization) [2]. The Rome IV criteria are nowadays the most widely used tools for diagnosing IBS and are based on the presence of recurrent abdominal pain on average at least 1 day/week in the last 3 months, being associated with two or more of the following criteria: symptoms related to defecation, change in frequency of stool, or change in the appearance of stool [3]. The Rome criteria further categorized patients into three subtypes: predominantly loose and frequent stools (IBS-D), infrequent and hard stools (IBS-C), and alternating diarrhea and constipation (IBS-M). A fourth group, IBS unclassified (IBS-U), was created to include patients who met the diagnostic criteria for IBS but whose bowel habits could not be reliably classified into one of the three previously mentioned groups [3,4]. Globally, IBS affects about 5–10% of the general population, with a substantial increase in the last decade, presenting a higher prevalence in young adults, particularly females, and in smokers [2]. Moreover, IBS could negatively affect one’s quality of life, increasing healthcare burdens and work absenteeism and reducing daily activity engagement [5].

Food and dietary habits seem to play a pivotal role in the management of patients with IBS, as around 85–90% report a worsening of symptoms in association with the consumption of certain foods [6,7]. Likewise, more severe gastrointestinal patient-reported symptoms are associated with concomitant food intolerances, highlighting the remarkable connection between food intolerances and IBS management [8].

Food intolerances are characterized by non-immunological gastrointestinal reactions triggered by the consumption of certain foods, leading to symptoms like abdominal pain, bloating, and changes in bowel habits [9]. Unlike food allergies, these intolerances often exhibit a dose-dependent pattern, where small amounts of the trigger food might be tolerated well, but larger quantities could lead to significant discomfort [10]. Consequently, patients with IBS often eliminate or substantially reduce their intake of trigger foods without any specific diagnostic testing and without clinical guidance, potentially leading to nutrient deficiencies [11].

The assessment of the impact of adverse food reactions on IBS presents a complex challenge, given the different food compositions and gastrointestinal physiology, hiding the identification of specific food items that may trigger or exacerbate IBS symptoms [12]. However, recent evidence focused the attention on dietary elimination and rechallenge studies, also suggesting that the symptom manifestation of IBS patients could be linked to their immunoglobulin status [13]. Moreover, recent studies suggested that increased epithelial barrier permeability may lead to immune activation and low-grade inflammation triggered by food antigens which could alter the motor and sensory gut functions in susceptible individuals [14].

In this context, the clinician’s approach involves a delicate balance between the complex and significant interplay between IBS and food intolerances, focusing on identifying and addressing the varied contributing factors for a holistic management of IBS patients, including an accurate diagnosis, effective patient communication, and the development of tailored therapeutic strategies [15].

This narrative review aims to thoroughly examine and synthesize existing research regarding the complex interplay between food intolerances and IBS. Various diagnostic approaches and treatment options will be discussed while emphasizing the role of dietary changes to facilitate an effective multidisciplinary approach to management.

## 2. IBS: Pathogenesis

IBS is a complex, multifactorial condition, and its pathogenesis is intricately linked with various gastrointestinal factors, including dysbiosis, mucosal leak, and biliary absorption alterations [16]. The background of these factors could be related to a genetic predisposition of affected patients that may influence individual susceptibility to IBS and the severity of symptoms, providing an explanation for the variability observed in IBS patients [17].

Dysbiosis, an imbalance in the gut microbiome, plays a pivotal role in the pathogenesis of IBS. This condition is characterized by a reduction in microbial diversity and a shift in the composition of the gut flora [18]. In particular, research has shown that patients with IBS often have lower levels of beneficial bacteria such as *Lactobacilli* and *Bifidobacteria*. These beneficial microbes play a crucial role in maintaining gut integrity, aiding digestion, and modulating the immune system [19]. Their reduction can lead to increased gut permeability, commonly known as “leaky gut”, and a heightened immune response, both of which are believed to contribute to the symptoms of IBS [20]. Additionally, an excess in Proteobacteria can lead to a depletion in the production of short-chain fatty acids (SCFAs), which are pivotal for maintaining mucosal integrity [21]. This imbalance can result from various factors such as diet, antibiotic use, infections, and stress. Furthermore, dysbiosis in IBS has been linked to increased gut sensitivity, contributing to the classic symptoms of IBS such as abdominal pain, bloating, and altered bowel habits [22].

Also, biliary absorption plays a role in IBS. Bile acids are essential for fat digestion and absorption, and alterations in bile acid metabolism have been observed in IBS patients. These alterations can lead to either an excess or a deficiency of bile acids in the colon, contributing to symptoms of diarrhea or constipation, respectively [23]. Furthermore, abnormal bile acid metabolism can affect the gut microbiome composition, promoting dysbiosis and perpetuating the cycle of symptoms [24].

Finally, increasing evidence also suggests that the central nervous system may play a pivotal role in modulating various gastrointestinal functions such as secretion, motility, and blood flow [25]. Conversely, signals originating from the gut are involved in regulating reflexes and the perception of gut events, involving both peripheral and central modulation [26]. Indeed, differences in brain responses to gastrointestinal stimuli have been documented in IBS patients. During rectal distention, IBS patients show greater activation in brain areas like the anterior cingulate cortex, amygdala, and dorsomedial frontal cortex compared with those with ulcerative colitis and healthy controls [27]. This suggests that individuals without IBS may have a more efficient activation of endogenous pain inhibition areas, possibly indicating a genetic predisposition to IBS [28].

In summary, IBS is a multifaceted disorder which arises from various causes, including genetic predisposition, microbiome changes, and immune responses [29]. Also, environmental factors such as food play a significant role in the pathogenesis of IBS by triggering gut sensitivity and motility issues. These factors collectively contribute to the broad spectrum of IBS symptoms and their variability, which can range from gastrointestinal discomfort to psychological manifestations like anxiety and fatigue [30]. A comprehensive understanding of these diverse mechanisms is essential for effectively addressing the intricate and varied aspects of IBS.

## 3. Adverse Food Reactions

Adverse food reactions can be broadly categorized into two main types: immune-mediated and non-immune-mediated [31]. Immune-mediated reactions, commonly known as food allergies, involve the immune system’s response to certain proteins in foods [32]. These reactions can range from mild symptoms to severe anaphylactic reactions, which can be life-threatening [33].

Celiac disease is an example of an immune-mediated, non-IgE adverse food reaction characterized by an autoimmune response to gluten that leads to intestinal damage and symptoms ranging from gastrointestinal distress to systemic issues like anemia [34,35]. Diagnosis typically involves serological tests and intestinal biopsy, while a gluten-free diet is the cornerstone of its management [36]. Recent evidence offers conflicting recommendations regarding the screening for celiac disease in subjects with IBS. While systematic screening for CD in all IBS patients is not recommended, a meta-analysis conducted by Ford et al. including 14 studies with 4204 individuals revealed a higher prevalence of celiac disease in IBS-D patients compared with the controls [37]. In this context, recent international guidelines suggest the possibility to perform serologic tests to rule out celiac disease, particularly in IBS-D patients [36,38].

Another immune adverse food reaction is nickel sensitivity, which is identified through a positive patch test for nickel [39]. Its typical clinical presentation is allergic contact dermatitis with a T-cell-mediated mechanism that arises from repeated skin exposure to nickel and triggers an immune response in individuals who are sensitive to this specific antigen [40]. Evidence suggests that sensitized subjects who ingested nickel could develop IBS-like gastrointestinal symptoms, in addition to characteristic cutaneous lesions [41,42]. In a study conducted by Rizzi et al., nickel susceptibility was higher in IBS patients compared with the healthy population, and a low-nickel diet improved gastrointestinal symptoms [41]. Furthermore, Wu et al. observed that the ingestion of dietary nickel could adversely affect the intestinal microbiota, specifically leading to a decrease in beneficial bacteria (i.e., *Lactobacillus*) [43]. Despite this evidence, the efficacy of a nickel-free diet remains a topic of debate, as consensus on its effectiveness has not been reached [44]. However, several studies have observed improvements in both dermatitis and gastrointestinal symptoms in patients following a nickel-free diet, highlighting its potential benefits in sensitized subjects [45,46,47].

On the other hand, non-immune mediated reactions, often referred to as food intolerances or sensitivities, do not involve the immune system [31]. These reactions are typically less severe and can include symptoms like digestive discomfort, headaches, or skin irritations [48]. Among these, lactose intolerance is a non-immune mediated adverse food reaction caused by a deficiency of the lactase enzyme, causing symptoms like bloating and diarrhea after lactose ingestion [49]. Hypolactasia manifests in three forms: primary, secondary, and congenital [50,51]. Primary lactase deficiency is common in adults and reflects a decline in lactase enzyme production [52]. Secondary deficiency, often reversible, arises from gastrointestinal conditions such as celiac disease, which damages the small intestine’s brush border [53,54]. Congenital lactase deficiency is the rare form and requires a lifelong avoidance of lactose [55]. Generally, lactose intolerance is frequently misdiagnosed as IBS due to the overlapping symptoms, although lactose-intolerant patients typically experience symptoms exclusively after ingesting products containing lactose [56]. The most reliable diagnostic technique for lactose intolerance is non-invasive hydrogen breath testing, and management of this condition requires a lactose-free diet or the administration of lactase enzyme supplements [57,58,59]. Therefore, it is useful to include lactose intolerance as a differential diagnosis in the evaluation of patients with suspected IBS, even though integration of the hydrogen breath test in the diagnostic protocol for IBS is still controversial [60].

The distinctive feature of these adverse food reactions lies in the fact that failing to eliminate the triggering food components not only impairs the patient’s quality of life but also affects the development and course of the disease, resulting in chronic digestive problems, severe intestinal damage causing malabsorption, and different systemic manifestations.

## 4. Which Foods and Food Patterns Can Cause Intolerance?

**Figure 1:**
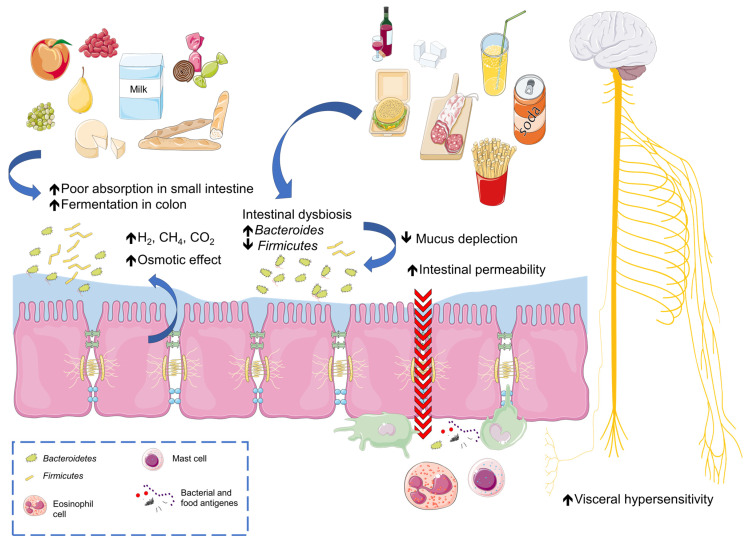
This figure illustrates the impact of high-FODMAP foods like fruits and milk (**left**) and processed foods (**right**) on gut health.

When exploring the variegated scenario of food intolerances, a complex condition emerges which is distinct from adverse food reactions but characterized by an important impact on patients’ health [48]. This condition primarily impacts the gastrointestinal tract but can also affect overall wellbeing [61]. To fully understand the nuances of food intolerances, it is important to consider the variety of foods and food patterns known to trigger adverse reactions and the underlying mechanisms of concomitant IBS. Figure 1 shows the interplay between foods and IBS pathophysiology.

### 4.1. Fermentable Oligo-, Di-, and Monosaccharides and Polyols (FODMAPs)

Fermentable oligo-, di-, and monosaccharides and polyols (FODMAPs) are a group of short-chain carbohydrates and sugar alcohols, including fructose, lactose, oligosaccharides (fructans, and galactans), and polyols such as maltitol, mannitol, sorbitol, and xylitol, first described in 2005 by Gibson et al. [62]. A food is classified as high in FODMAPs if it contains more than 4 g of lactose, over 0.2 g of excess fructose, or more than 0.3 g of other FODMAPs in a single serving [63].

However, the dietary content of FODMAPs varies significantly across different geographical regions, as it is influenced by the varying levels of these compounds in local diets [64]. Notably, fructose and fructans are the most prevalent FODMAPs among Mediterranean countries [65], as the Mediterranean diet is rich in vegetables, fruit, and legumes [66].

The gastrointestinal mechanism of FODMAPs is based on their incomplete absorption in the small intestine, which leads these carbohydrates to transit into the large intestine, where they exert osmotic activity, attracting water into the intestinal lumen [67]. In the colon, these unabsorbed carbohydrates undergo fermentation by the gut microbiota, producing gases including hydrogen, methane, and carbon dioxide. This process is implicated in the pathogenesis of common symptoms associated with IBS, such as bloating, abdominal discomfort, and altered bowel habits [13]. The osmotically driven increase in luminal fluid, coupled with gas production, contributes to intestinal distension that could amplify the perception of bloating and pain [68].

Recent evidence suggests that fermentation and osmotic action could not fully provide an explanation for IBS intolerance to FODMAPs. Some authors hypothesized a possible loss of local tolerance to food triggered by infection as a mechanism underlying abdominal pain in IBS. A study on mice exposed to a food antigen during infection showed increased sensitivity, and they developed visceral hypersensitivity. This response was linked to local production of specific IgE antibodies and the activation of mast cells. Histamine, released by mast cells, was found to increase pain signaling by affecting specific neural pathways. The prevention of visceral hypersensitivity was possible by either stabilizing the mast cells or using an IgE-blocking antibody [69]. Consistently, clinical tests on IBS patients revealed a similar response to certain dietary compounds containing FODMAPs, with immediate mucosal reactions and increased mast cell activity [70]. These pathways may be enhanced by increasing mucosal permeability caused by dysbiosis and mucus depletion, which favor bacterial and food antigen translocation [71].

### 4.2. Fructose Malabsorption

Among FODMAPs, fructose is a simple sugar that can be investigated independently, unlike other FODMAPs, because of its unique absorption mechanism in the body and the several metabolic pathways in which it is involved [72]. Additionally, it can be found in large amounts in fruits, honey, and syrups and is often added to several processed foods in the Western diet as sucrose or high fructose corn syrup, resulting in its consistent consumption over the past few decades [73].

High fructose intake can strain the absorptive capacity of the small intestine, potentially resulting in fructose malabsorption even among subjects without hereditary fructose intolerance [74]. Fructose malabsorption involves the inability to effectively absorb fructose due to a deficiency in fructose transporters within the small intestine and may lead to the accumulation of unabsorbed fructose in the colon [75]. This process leads to an osmotic effect enhanced by fermenting metabolism of colonic bacteria, with a consequent production of gas. Thus, the main symptoms involved are bloating, abdominal pain, flatulence, and diarrhea [76]. Furthermore, individuals with fructose malabsorption are predisposed to increased gastrointestinal sensitivity, which might exacerbate IBS symptoms [77].

Fructose malabsorption is a condition that can be assessed using hydrogen or methane breath tests. The standard procedure involves the ingestion of 25 g of fructose, followed by monitoring for increases in hydrogen and methane in the breath. This approach is based on the principle that undigested fructose ferments in the intestine, producing these gases [78]. This condition appears to be more prevalent in patients with IBS, affecting about 22% of this group, which is a higher rate compared with the healthy population. Moreover, the prevalence of fructose malabsorption does not seem to vary significantly across different subtypes of IBS [77].

On the other hand, the diagnosis of fructose malabsorption in patients with IBS is complicated by the frequent overlap with small intestinal bacterial overgrowth (SIBO). A study conducted by Jung et al. in 2019 [79] was particularly notable because it excluded patients with SIBO to assess the potential relationship more accurately between fructose malabsorption and IBS. The findings revealed that fructose malabsorption was significantly more common in SIBO-negative IBS patients compared with the asymptomatic controls. This suggests a potential association between fructose malabsorption and IBS [79].

These findings collectively highlight the significance of fructose malabsorption in the context of IBS and the need for more detailed guidelines on dietary management for those affected by this condition.

### 4.3. Non-Celiac Gluten Sensitivity

Non-celiac gluten sensitivity (NCGS) is a condition characterized by intestinal and extraintestinal symptoms related to the ingestion of gluten-containing foods in individuals who do not have celiac disease or a wheat allergy [80]. Despite its distinct clinical condition, NCGS exhibits symptomatologic analogisms with these latter conditions [81]. Consequently, it is addressed in this section, particularly because its management necessitates a dietary approach involving either complete or partial exclusion of gluten, which represents the main storage protein in wheat grains [82]. In NCGS, the consumption of gluten leads to a range of symptoms, such as abdominal pain, bloating, bowel habit alterations, and even non-gastrointestinal symptoms like headache, fatigue, and joint pain [80].

Unlike celiac disease, NCGS does not have a clear biomarker or intestinal damage evident in diagnostic tests [83]. However, the symptoms significantly improve or resolve upon following a gluten-free diet [84]. This elimination is currently the main method for diagnosing NCGS [80]. The prevalence of NCGS is still under investigation, but it appears to be more common than celiac disease, affecting a broader spectrum of the population [85]. Its pathophysiology is not fully understood, but it is believed to involve an innate immune response [86]. This contrasts with celiac disease, which involves an adaptive immune response with autoimmunity [87]. Furthermore, the role of FODMAPs in NCGS symptoms is also a subject of ongoing research, as many gluten-containing foods are also high in FODMAPs [88]. Given the overlap of symptoms with other gastrointestinal disorders, particularly IBS, NCGS is often a diagnosis of exclusion [89]. Careful differential diagnosis is crucial to distinguish NCGS from celiac disease, a wheat allergy, and other gastrointestinal disorders.

A recent study conducted by Ahmed et al. identified 12.4% of IBS patients having biological evidence of NCGS [90]. This was supported by a double-blind, placebo-controlled trial by Biesiekierski et al. involving 34 IBS patients. This study found that 68% of those consuming gluten reported uncontrolled symptoms versus 40% in the gluten-free group. Patients on a gluten-free diet reported better improvement in gastrointestinal symptoms like pain, bloating, and tiredness. This suggests that gluten sensitivity may be a distinct condition in some IBS patients [91]. In contrast, the same authors conducted a study on 37 individuals with NCGS and IBS. They first followed a low-FODMAP diet and then were given diets high in gluten, low in gluten, or a control diet in a double-blind crossover trial. Only 8% of the participants showed gluten-specific effects, and no significant changes were noted in the biomarkers. This study suggests that symptoms in NCGS patients may not be specifically or dose-dependently related to gluten [92].

In summary, the ambiguity in clinical presentations and responses to dietary interventions underscores the need for further research to better understand and manage NCGS.

### 4.4. Histamine

Histamine is a compound found in many foods such as cheeses, fermented products, and specific fish types [93], and it has recently been recognized as a potential trigger for IBS symptoms [94]. It is well known that a deficiency in diamine oxidase, the enzymes able to metabolize histamine, can lead to a toxic accumulation of this metabolite, mimicking an allergic reaction with symptoms like headaches, rashes, and gastrointestinal distress [95].

In the context of IBS, it was hypothesized that histamine might contribute to symptom exacerbation by triggering an immune response or altering gut motility and sensitivity. This mechanism has not yet been elucidated, but it was hypothesized that the intestinal microbiota composition plays a pivotal role, particularly in the context of IBS [96]. Microorganisms utilize decarboxylation reactions for histamine production, a strategy aiding their survival by reducing environmental pH and providing an alternative energy source. Bacterial diamine oxidase contributes to producing ammonia and hydrogen peroxide [97]. Patients affected by this condition often exhibit increased *Proteobacteria* levels in feces, which are responsible for a reduction in intestinal microbiota diversity and intestinal barrier permeability [98]. In particular, species like *Klebsiella aerogenes*, which have high levels of bacterial histidine decarboxylase, convert dietary histidine into histamine, especially when there is a high consumption of fermentable carbohydrates. On the other hand, an increased presence of *Lactobacilli* can decrease histamine production due to their ability to lower the pH in the gut through lactic acid production. This bacterially generated histamine can activate histamine receptors on mast cells, which is a significant factor in the development of visceral hypersensitivity [99].

### 4.5. Food Additives

Food additives are an integral part of the modern human Western diet and are gaining attention for their potential health impacts [100]. Currently, there is limited data on the effects of food additives on the gut microbiota in IBS patients. Moreover, most existing studies on the effects of food additives on gut health have been conducted in animals, indicating a need for more human research [101]. Evidence suggests that artificial sweeteners, emulsifiers, and food colorants may be triggers of IBS via their impact on gut microbiota. Nonetheless, it was hypothesized that these additives could induce dysbiosis, leading to changes in the gut barrier and immune response activation [102].

Studies in rats and mice have shown that non-caloric sweeteners can alter gut microbiota composition, increasing certain bacteria like *Bacteroidetes* and decreasing others like Firmicutes [103,104]. Human studies are limited but suggest that these sweeteners might reduce bacterial diversity without necessarily increasing bacterial abundance. In this context, emulsifiers found in processed foods can change gut microbiota and potentially increase intestinal inflammation and permeability, while food colorants like titanium dioxide may also alter gut microbiota, but their long-term impact on humans is not well understood [105,106]. The role of these additives in conditions like IBS is an emerging area of research, highlighting the need for further studies to understand their effects on gut health and disease.

### 4.6. Other Foods Identified as Triggers in IBS Patients

Aside from FODMAPs, histamine, and additives, a wide range of other food components may also trigger adverse symptoms, misleading intolerances in patients with IBS. These components mainly include specific proteins in legumes and nuts as well as naturally occurring chemicals present in a broad spectrum of fruits and vegetables. Their impact on the gastrointestinal tract is multifaceted, ranging from direct irritation of the gut lining to modifications in gut motility and changes in the composition and growth of gut bacteria [107].

One significant factor in food intolerance is caffeine. In some IBS patients, particularly those with IBS-D, caffeine can increase gastrointestinal motility, exacerbating symptoms. Caffeine acts as a natural laxative by increasing peristalsis, resulting in diarrhea and abdominal discomfort, hallmark symptoms of IBS-D [108]. Moreover, caffeine stimulates the release of gastrin, a hormone that can increase gastric acid secretion, potentially leading to gut irritation and discomfort [109]. Additionally, this ammine can affect the enteric nervous system, causing an alteration of pain perception and increasing sensitivity of the gut lining, further contributing to discomfort in IBS patients. It is also important to note that caffeine’s diuretic effect can lead to dehydration, which in turn can aggravate IBS symptoms [110]. In a study conducted by Koochakpoor et al., a higher coffee and caffeine intake was associated with increased odds of IBS in adults. Additionally, higher caffeine consumption correlated with greater severity of IBS symptoms in overweight subjects [111].

Spicy foods, especially those containing capsaicin like hot peppers, represent another dietary concern for IBS patients [112]. As pointed out by Böhn et al. in 2013, these foods can irritate the gastrointestinal tract by activating the transient receptor potential vanilloid 1 channel. These channels, present in the gut, play a key role in sensing heat and pain. When activated by capsaicin, they can lead to an increased sensation of pain and discomfort, often exacerbating IBS symptoms [113].

In addition, a significant proportion of patients with IBS attributed a worsening of their symptoms to high-fat foods, leading many of them to avoid such foods for symptom relief [114]. Basic science suggests that duodenal lipids can disturb small bowel motility and gas clearance, causing bloating and enhancing colorectal hypersensitivity in IBS patients [13,115]. However, strong evidence linking dietary fat to IBS is limited, with no conclusive clinical trials supporting reduced fat intake for symptom improvement [116]. Contrarily, some studies suggest benefits from dietary fat in IBS, especially polyunsaturated fatty acids, which could mitigate intestinal inflammation [117]. On the other hand, high-fiber diets may also have significant implications for IBS [118]. Fiber is typically recommended for its health benefits, such as improving bowel regularity and overall gut health [119]. However, its effects on individuals with IBS can vary considerably. The type of fiber consumed, whether soluble or insoluble, along with the individual tolerance should be considered among patients with IBS. Soluble fiber dissolves in water and forms a gel-like substance in the gut which can help to soften stools and promote smoother bowel movements, which can be beneficial in IBS-C patients [120]. However, in some cases, excessive intake of soluble fiber may lead to gas and bloating, potentially worsening IBS symptoms. Insoluble fiber can be advantageous for regular bowel movements but might exacerbate symptoms in individuals with IBS-D due to its laxative effect and rapid transit through the gastrointestinal tract [118].

Finally, alcohol is another important dietary factor to consider in the management of IBS, although evidence is still limited. Given that alcohol is primarily absorbed in the upper intestinal tract, its impact on the small intestine and colon may be associated with its oxidative and nonoxidative metabolism, being able to alter tissue homeostasis and leading to chronic intestinal inflammation [121]. In this field, the specific alcohol interaction with the central nervous system via the gut-brain axis remains unclear, but studies propose that alcohol-triggered leaky gut may result in a systemic inflammation affecting neuronal function [122]. This condition could contribute to symptoms like anxiety during alcohol withdrawal [123], and even moderate alcohol consumption can lead to increased gut motility and disrupt the balance of gut microbiota, potentially developing symptoms like diarrhea, bloating, and abdominal pain [124,125].

## 5. Which Dietary Patterns Are Useful in Food Intolerance in IBS Patients?

Dietary and lifestyle changes are often recommended for subjects with IBS due to several mechanisms like the direct influence of food, changes in gut microbiota, and immune reactions that could cause symptoms. While there is increasing interest in exclusion diets as a treatment strategy for IBS, the evidence regarding their effectiveness is lacking. The 2023 Italian guidelines for the management of IBS recommend a dietary approach based on so-called “traditional dietary advice” developed by the National Institute for Health and Care Excellence (NICE) and the British Dietetic Association (BDA) as first-line treatment [38,116]. Traditional dietary advice emphasizes the role of lifestyle modification, physical activity, and dietary management in effectively controlling the symptoms of IBS. Individuals with IBS are advised to proactively improve leisure and relaxation time while also focusing on eating behavior. This includes recommendations for regular meals, allowing sufficient time to eat, and adopting a relaxed eating approach. Additionally, such dietary treatment addresses the limitation of certain foods often associated with gastrointestinal discomfort in IBS patients. These include spicy foods, alcohol, tea, coffee, and foods high in fats and insoluble fibers, with a suggestion to increase the intake of soluble fibers [38].

However, these recommendations are largely based on moderate-to-low-quality evidence from randomized controlled trials and controlled studies or on clinical expertise. These issues highlight an unmet need for more robust research to further validate and refine the management strategies for IBS through lifestyle and dietary changes. In this field, several authors have suggested a variety of dietary interventions to customize nutritional therapy for patients suffering from IBS. Table 1 summarizes the various dietary patterns proposed and their impact on IBS symptoms.

### 5.1. Low-FODMAP Diet

The low-FODMAP diet is a dietary approach designed to reduce symptoms associated with IBS, involving a restriction of foods high in FODMAPs such as certain fruits, vegetables, grains, dairy products, and sweeteners as they can ferment in the gut and cause bloating, gas, abdominal pain, and other gastrointestinal symptoms. This dietary approach has demonstrated efficacy in providing gastrointestinal symptom relief for around 70% of subjects with IBS [132]. A recent meta-analysis conducted by van Lanen et al., that evaluated 772 subjects highlighted the positive effect of the low-FODMAP diet on gastrointestinal symptoms and quality of life in IBS subjects compared with the control diets, such as the habitual diet, Mediterranean diet, or a typical diet specific to the country where the study was conducted [133].

The low-FODMAP diet consists of a three-phase approach which has a “top-down” administration to identify food triggers and manage symptoms related to dietary sensitivities. Phase 1, lasting 4–6 weeks, involves the strict avoidance of all high-FODMAP foods. Phase 2, the rechallenge phase, sees high-FODMAP foods gradually reintroduced into the diet and enables patients to identify specific food triggers. Lastly, phase 3 implicates the creation of a long-term, customized FODMAP diet plan based on individual tolerances identified in earlier phases, aiming to establish a personalized and sustainable dietary approach [134].

Every step of the three-phase “top-down” low-FODMAP diet should be individually customized. In this context, the dietitian’s expertise is essential not only for avoiding nutritional deficiencies and maintaining a balanced diet but also to prevent any loss of adherence to the diet plan [135].

On the contrary, the “bottom-up” approach is an alternative administration of a low-FODMAP diet which involves the limitation of just one or two FODMAP subgroups, considering the individual’s dietary history and ethnic risk factors [134]. This strategy could be more appropriate for those with less severe symptoms, in children, or in patients at risk of malnutrition or predisposed to eating disorders [136].

Overall, the top-down low FODMAP approach, although initially more challenging, may lead to greater long-term success in identifying specific food triggers [137]. This is particularly valuable for patients with uncertain FODMAP tolerance, infrequent FODMAP consumption, or those experiencing severe symptoms, potentially resulting in more accurate and effective long-term dietary management [136].

While the low-FODMAP diet is often recommended for patients with IBS, its practical implementation presents several challenges, and the long-term effects on gut microbiota are not fully understood [138]. Considering its complexity, along with potential nutritional deficiencies and the risk of developing restrictive eating habits that necessitate the supervision of a dietician, the low-FODMAP diet is recommended as a second-line approach [38]. In fact, despite its effectiveness at managing symptoms, the low-FODMAP diet may not be a universally suitable or sustainable option for all individuals with IBS [139].

### 5.2. Gluten-Free Diet

The gluten-free diet, which is originally designed for patients with celiac disease, has gained attention as a potential dietary approach for managing symptoms of IBS [140]. However, evidence has shown different results regarding the efficacy of this treatment in IBS, and several randomized controlled trials (RCTs) provide a picture that is still conflicting [141]. Recently, Arabpour et al. conducted a meta-analysis on nine RCTs and reported that the gluten-free diet is not effective enough to be recommended for IBS patients, and its efficacy is significantly lower than that of the low-FODMAP diet [142]. The variability in the effectiveness of a gluten-free diet could depend on the placebo or nocebo individual effect and differences in sensitivity to gluten or fructans. In fact, a gluten-free diet may inadvertently lead to a reduction in FODMAP intake, thus contributing to symptom improvement [143].

A critical issue of the gluten-free diet is the implication of several dietary modifications which can impact the nutritional status. Given that whole grains rich in gluten are also sources of fiber, vitamins, and minerals, patients who reduce their intake of these grains may thereby decrease their consumption of carbohydrates and fiber [144]. Furthermore, gluten-free products frequently have increased amounts of sugar, saturated fats, and sodium and tend to be more expensive compared with their gluten-containing equivalents [145].

We must note that the effects of a gluten-free diet on the gut microbiota in individuals with IBS remain unclear. Nonetheless, analogous to the low-FODMAP diet, it is known that avoiding gluten predominantly influences bacterial species that metabolize carbohydrates and starch for energy [146]. A recent study observed that a gluten-free diet can lead to a decreased presence of bacterial strains that exert a protective effect in individuals with IBS, such as Actinobacteria [147]. Conversely, Hansen et al. indicated that even minimal gluten intake can significantly alter the gut microbiota, particularly by reducing the *Bifidobacteria* count in non-celiac individuals on a low-gluten diet [148]. In summary, the current evidence does not support the adoption of a gluten-free diet for subjects suffering from IBS [38].

### 5.3. Lactose-Free Diet

The administration of a lactose-free diet, initially tailored for lactose intolerance, has been proposed to approach patients with IBS and symptoms associated with lactose intake [56]. The rationale behind this approach is that the primary cause of IBS symptoms often lies in undigested lactose passing into the small and large intestines [13]. This diet requires the exclusion of lactose, a disaccharide sugar mainly found in dairy products which can effectively be classified as a FODMAP [130]. Although some patients with IBS often benefit from a lactose-free diet, the evidence is still inconsistent and shows conflicting results [130,149]. Nevertheless, the presence of lactose in medications seems not to influence their effectiveness or induce gastrointestinal symptoms compared with lactose-free formulations, meaning that no drugs should be avoided in the design of a lactose-free diet [150].

Moreover, the nutritional implications of a lactose-free diet are critical, as dairy products are major sources of calcium, vitamin D, and proteins. Thus, adherence to a lactose-free diet without adequate dietetical management could lead to deficiencies in these nutrients [151]. Additionally, lactose-free diets may alter the gut’s microbial composition, potentially affecting the fermentation of carbohydrates and the production of short-chain fatty acids [152]. In summary, when considering a lactose-free diet, IBS patients should be informed about the limited high-quality evidence supporting its effectiveness at alleviating symptoms. It should be recommended only for patients with a positive lactose hydrogen breath test confirming lactose intolerance.

### 5.4. Mediterranean Diet

The Mediterranean diet is characterized by a substantial intake of plant-based foods such as fruits, vegetables, whole grains, legumes, and nuts along with olive oil and a moderate consumption of fish and dairy products [153]. Evidence shows that lower adherence to the Mediterranean diet correlates with a higher incidence of IBS [154], while in a study conducted by Paduano et al., a Mediterranean balanced diet was able to improve quality of life and was more appreciated by patients compared with the low-FODMAP diet and gluten-free diet [128]. However, Chen et al. observed that some typical food of this dietetic pattern may exacerbate symptoms in some individuals [154]. For example, the high fiber intake which characterized this pattern of diet might exacerbate symptoms in patients with IBS-D, even though a large amount of fiber may be beneficial for patients with IBS-C [118].

Thus, the Mediterranean diet could be a feasible and potentially therapeutic dietary intervention for managing both gut and psychological symptom burdens in IBS, but it is important to consider that this diet should be personalized considering the various spectrum of individual symptoms [155]. A final consideration regarding the Mediterranean diet pertains to its beneficial effects across various aspects of human health, such as cardiovascular disease prevention, metabolic health, and cognitive function. This diet, rich in heart-healthy fats, antioxidants, and fiber, has been shown to lower the risk of heart disease, stroke, and type 2 diabetes [156]. In brief, the Mediterranean diet stands as a balanced dietary approach not only for managing symptoms in conditions like IBS but also for promoting overall health.

### 5.5. Other Dietary Patterns for the Management of Food Intolerance in IBS Patients

The assessment of other dietary strategies and patterns is in continuous evolution, particularly in the context of managing food intolerance in IBS patients. For example, the advice to reduce the fiber content in a diet could be a good strategy for patients with IBS-D [118], and a low-fat diet could be suggested for reducing IBS symptoms related to gut motility [107], but both suffer from a lack of RCTs and misleading long-term health impacts [157]. Another strategy adopted to mitigate abdominal pain and diarrhea could be the reduction of high-fructose foods [72]. Similarly, the low-histamine diet is hypothesized to alleviate IBS symptoms by reducing histamine intake [94], but both of these dietary strategies lack extensive validation in the context of IBS [157]. Finally, the Paleolithic diet, which eliminates common IBS triggers like processed foods, grains, and dairy products, is an obvious example where the perceived benefits are predominantly based on subjective experiences and social media rather than scientific evidence [158].

This lack of evidence highlights the crucial role of a skilled dietitian for adopting a personalized and evidence-based dietary intervention for IBS patients.

## 6. Future Prospective and Unmet Needs

The management of IBS and food intolerances is shaped by the evolving understanding of these conditions and the necessity for a more personalized approach to treatment. A key aspect of this evolution involves a multidisciplinary team, encompassing dietitians, gastroenterologists, and psychologists who collaborate to devise a thorough treatment strategy.

In the field of pathophysiology, a significant area of interest and a potential breakthrough is the growing role of gut microbiome research, which promises to uncover new insights into the pathophysiology of IBS and novel therapeutic targets. Several interventions aimed to modulate the gut microbiome, such as prebiotics, probiotics, dietary modifications, and fecal microbiota transplantation, offering effective opportunities for treatments targeting the causes of IBS rather than exclusively addressing the symptoms. Indeed, the gut microbiome influences nutrient metabolism in different ways [159]. Firstly, the gut microbiota is involved in the production of SCFAs, which are involved in maintaining gut barrier function [160]. Nevertheless, the relationship between SCFAs and IBS is intricate, with different fecal SCFA levels reported across different IBS subtypes [161]. Another significant contributor to nutrient metabolism influenced by the gut microbiome is tryptamine, a metabolite of tryptophan [162]. Tryptamine influences colonic function and transit through its interaction with serotonin receptors, and elevated tryptamine levels have been observed in IBS-D, suggesting its involvement in altered gut motility [163]. Furthermore, lipopolysaccharides derived from gram-negative bacteria have been involved in IBS-D, as a high-FODMAP diet has been associated with increased fecal lipopolysaccharides levels, exacerbating barrier dysfunction and hypersensitivity [164]. Additionally, the gut microbiome could modulate immune activation in IBS, as supported by the presence of low-grade inflammation and altered cytokine profiles in affected subjects [165]. However, there remains a substantial gap in understanding exactly how changes in the gut microbiome directly affect gut function and the manifestation of IBS symptoms.

Another critical aspect is the exploration of genetic and epigenetic factors in IBS and food intolerances [166]. While there is evidence of a genetic predisposition to these conditions, the specific genetic markers and how they influence treatment response are still unclear [167]. This gap in knowledge is a significant barrier to the development of personalized medicine approaches, which could lead to more effective and tailored treatment strategies.

A central challenge is further compounded by the difficulty in diagnosing IBS, as it often involves overlapping symptoms with other gastrointestinal disorders and lacks a definitive diagnostic test. This issue, coupled with the variability in dietary triggers among individuals, makes the relationship between IBS and food intolerances like lactose intolerance or non-celiac gluten sensitivity particularly challenging to manage. It is known that the identification of food intolerances and allergies in IBS has focused on IgE-mediated response. However, this latter mechanism appears to be unclear in the context of IBS [8]. Actually, adverse food reactions in IBS may involve an alternative immunological mechanism, potentially mediated by IgG antibodies. An RCT conducted by Atkinson et al. suggested that food elimination based on IgG antibodies could be effective at reducing IBS symptoms [168]. In addition, Vita et al. observed that increased levels of food-specific IgG antibodies may co-occur with elevated concentrations of intestinal permeability biomarkers, and these findings could be driven by commonly highly reactive foods, suggesting the possibility to consider food sensitives in the assessment of the intestinal permeability in the context of IBS [169]. Nevertheless, the role of IgG antibodies remains a topic of debate, as they are also found in healthy individuals [168]. Thus, IgG assessment in IBS is not yet recommended by the international scientific societies [170,171].

Patient education and treatment adherence are also critical in managing IBS and food intolerances. The effectiveness of dietary interventions, such as the low-FODMAP diet, is often limited by the patient’s adherence to these complex dietary changes over time. The psychological impact of dietary restrictions, potentially leading to food fears and eating disorders, is an often-overlooked concern in treatment plans.

Looking toward the future, there is hope for significant advancements in understanding and managing IBS and food intolerances. The integration of digital health technologies, such as mobile health applications, telehealth, and wearable devices, presents exciting prospects for more personalized, real-time, and accessible care for patients [172]. These technologies could revolutionize patient education, symptom tracking, and dietary management [173].

## 7. Conclusions

The management of IBS and food intolerances still presents substantial challenges in gastroenterology and dietetics settings, although several gaps still exist in the overall management.

IBS is widely recognized as a multifactorial disorder influenced by several factors, including diet, stress, microbiota, genetics, and gastrointestinal motility. Its pathogenesis has not been comprehensively elucidated by the scientific community. In fact, while most of the evidence suggests that IBS represents the primary condition and that food intolerances arise as a consequence of intestinal alterations in IBS patients, some studies propose that food intolerances may significantly contribute to the onset of IBS symptoms. According to this perspective, the ingestion of specific foods may trigger adverse reactions in the gastrointestinal tract, contributing to inflammation and hypersensitivity.

Despite the existing evidence, IBS remains a condition defined largely by its symptoms rather than its causes, potentially triggered by different dietary factors which could interact with other features distressing the intestinal pathophysiology, including dysbiosis and mucosal leaks. In this context, it is crucial to accurately define a diagnostic process for food intolerances, as the symptomatic overlap of IBS with other gastrointestinal disorders frequently results in misdiagnosis or delayed diagnosis. Complexities in managing IBS and food intolerances are amplified by issues related to treatment compliance and patient education. The placebo and nocebo effects, which are frequently influential in dietary intervention studies, further complicate the interpretation of results. Misinformation and myths surrounding the condition of IBS are extensive and often worsened by the adoption of non-evidence-based dietary changes which are not only potentially ineffective but also harmful, possibly leading to nutritional deficiencies.

## Figures and Tables

**Table 1:** Different dietary patterns and their impact on IBS symptoms.

FOODS	MECHANISM OF ACTION	ASSOCIATION WITH IBS	SPECIFIC DIETARY APPROACH
FODMAPs [126]	Poor absorption in small intestine, causing osmotic effect Fermentation in colon, producing gas and attracting water.	High prevalence in IBS → 33% of patients with functional gastrointestinal disorders, including IBS Symptoms associated: Triggers bloating, abdominal discomfort, and altered bowel habits	Low-FODMAP diet to identify triggers: ✓ Around 60% of IBS patients have symptom relief ✕ Alteration of gut microbiota not fully addressed ✕ Inadequate nutrient intake due to the restrictive nature of the diet and risk of restrictive eating habits
Fructose [77]	Malabsorption due to deficiency in fructose transporters Leads to fermentation in colon	Common in IBS patients → The prevalence of fructose intolerance in patients with IBS is about 22% Symptoms associated: Causes bloating, abdominal pain, flatulence, and diarrhea	Fructose-restricted diet (FRD): ✓ FRD significantly reduced abdominal pain and discomfort, bloating, and stool frequency, whereas no change was observed for stool consistency ✕ Inadequate nutrient intake due to the restrictive nature of the diet and risk of restrictive eating habits
Non-Celiac Gluten Sensitivity [127,128]	Symptoms triggered by gluten without celiac antibodies, villous atrophy, or allergies	Prevalence of NCGS in IBS → between 23 and 49% Symptoms associated: Intestinal and extra-intestinal symptoms (i.e., altered bowel habit, abdominal pain, bloating, headache, fatigue, and joint pain)	Gluten-free diet: ✓ Reduction in gastrointestinal symptom severity like bloating and abdominal pain ✕ Decreased consumption of carbohydrates and fiber and higher intake of sugar, saturated fats, and sodium. Gluten-free products are more expensive than their gluten-containing equivalents.
Lactose [129,130]	Incomplete digestion due to lactase deficiency Fermentation in colon	Misdiagnosed as IBS → 60.7% of the patients with IBS and 43.5% in the control group Symptoms associated: Triggers bloating and diarrhea after ingestion	Lactose-free diet: ✓ Decrease in abdominal pain, bloating, and diarrhea in 59.1% of IBS patients ✕ Deficiencies of calcium, vitamin D, and proteins and inadequate intake of calcium, vitamin D, and proteins
Food Additives [131]	Alteration of gut microbiota Induction of inflammation and permeability changes	Potential trigger of IBS onset → Between 1 and 2% of general population, with no direct data on IBS Symptoms associated: Effects on gut health are ongoing	Avoidance of artificial sweeteners, emulsifiers, and colorants: ✓ Reduction in processed food intake, typically present in the Western diet ✕ No data on IBS available

## Data Availability

Not applicable.
